# Modulation of Interleukin-12 activity in the presence of heparin

**DOI:** 10.1038/s41598-017-05382-1

**Published:** 2017-07-13

**Authors:** Srinivas Jayanthi, Bhanu prasanth Koppolu, Khue G. Nguyen, Sean G. Smith, Barbara K. Felber, Thallapuranam Krishnaswamy Suresh Kumar, David A. Zaharoff

**Affiliations:** 1 Department of Chemistry and Biochemistry, University of Arkansas, Fayetteville, AR USA; 2 Department of Biomedical Engineering, University of Arkansas, Fayetteville, AR USA; 3 Joint Department of Biomedical Engineering, North Carolina State University and the University of North Carolina-Chapel Hill, Raleigh, NC USA; 4 Cell and Molecular Biology Program, University of Arkansas, Fayetteville, AR USA; 5 Department of Microbiology and Immunology, University of North Carolina, Chapel Hill, NC USA; 6 Human Retrovirus Pathogenesis Section, Vaccine Branch-National Cancer Institute, Frederick, MD United States

## Abstract

Glycosaminoglycans (GAGs), especially heparin and heparan sulfate (HS), modulate the functions of numerous cytokines. The aims of this multidisciplinary research were to characterize heparin binding to interleukin-12 (IL-12) and determine the mechanism(s) by which heparin influences IL-12 bioactivity. Heparin and HS were found to bind human IL-12 (hIL-12) with low micromolar affinity and increase hIL-12 bioactivity by more than 6-fold. Conversely, other GAGs did not demonstrate significant binding, nor did their addition affect hIL-12 bioactivity. Biophysical studies demonstrated that heparin induced only minor conformational changes while size-exclusion chromatography and small angle X-ray scattering studies indicated that heparin induced dimerization of hIL-12. Heparin modestly protected hIL-12 from proteolytic degradation, however, this was not a likely mechanism for increased cytokine activity *in vitro*. Flow cytometry studies revealed that heparin increased the amount of hIL-12 bound to cell surfaces. Heparin also facilitated hIL-12 binding and signaling in cells in which both hIL-12 receptor subunits were functionally deleted. Results of this study demonstrate a new role for heparin in modulating the biological activity of IL-12.

## Introduction

Polyanionic glycosaminoglycans (GAGs) have been shown to bind numerous growth factors and cytokines^1–5^. The physiological significance of this binding is two-fold. First, GAGs can serve as co-receptors on cell surfaces to maintain high, local concentrations of cytokines^6–10^. Second, GAGs can regulate bioactivities of growth factors and cytokines through multiple mechanisms including dimerization and protection from proteolytic degradation^11–14^. The two most heavily *N*-sulfated GAGs, heparin and heparan sulfate (HS), are known to interact with more than 400 proteins involved in various biological processes^10^. In its most obvious role, heparin binds to antithrombin and increases its anticoagulant activity. Heparin binding is also an essential prerequisite for basic fibroblast growth factor (bFGF) dimerization and engagement with its high affinity receptor for angiogenesis, wound healing, stem cell differentiation, etc.

Beyond antithrombin and bFGF, the binding of sulfated GAGs plays a significant role in the biology of numerous other pleiotropic cytokines and growth factors^15–18^. For example, binding of GM-CSF to sulfated GAGs in the extracellular matrix produced by stromal cells is critical for maintaining high local concentrations in the bone marrow microenvironment^19^ and increasing GM-CSF-induced myeloid cell proliferation^20^. Additionally, heparin binding decreases interferon-γ (IFN-γ) clearance^21^, interferes with cellular binding^22^ and inhibits IFN-γ-induced upregulation of class II MHC and adhesion molecules on the surfaces of vascular endothelial cells^15, 23^.

GAG-interleukin interactions have been explored, albeit to a significantly lesser extent. Interleukins mediate signaling primarily between leukocytes. Given that leukocytes are highly mobile populations of cells, the need for compartmentalization of interleukins through GAG binding is not as obvious. Nevertheless, heparin and HS have been shown to bind to many interleukins with mixed effects on their bioactivities.

Heparin binds to human but not murine IL-2^24, 25^, however, heparin binding has no effect on hIL-2 bioactivity as measured by proliferation of CTLL-2 cells^25^. The effect of heparin/HS binding on IL-3 bioactivity is concentration-dependent with IL-3-induced proliferation of myeloid cells increasing at low heparin concentrations and decreasing at high heparin concentrations^20^. HS binds to and inhibits IL-4-based suppression of lipopolysaccharide-stimulated monocytes^26^. Yet, HS enhances the proliferative activity of IL-5 in specialized Baf-IL-5 cells^27^. Heparin/HS has no impact on IL-6-stimulated proliferation of Ba/F3 cells^28^. Heparin complexation protects IL-7 from proteolytic degradation, however, heparin binding inhibits the growth of IL-7 dependent pre-B cells^29^. HS augments IL-8-induced neutrophil chemotaxis and Ca^2+^ responses^30^ while heparin facilitates similar IL-8-induced Ca^2+^ responses but either inhibited, or had no impact on, neutrophil chemotaxis^30, 31^. Lastly, heparin and HS both inhibit IL-10-induced upregulation of CD16 and CD64 on monocytes/macrophages^13^. In general, while the effects of heparin/HS on interleukin bioactivity are mixed, interleukin binding to highly sulfated GAGs appears to be more inhibitory than augmentative.

IL-12 has been shown previously to be a heparin-binding protein^32, 33^. Our recent *in silico* analysis found two heparin-binding domains located on the p40 subunit of IL-12^33^. These sites were exploited in the single-step purification of tagless IL-12, while the affinity of heparin for IL-12 was well characterized via isothermal calorimetry (ITC)^33^.

In this study, the ability of heparin/HS to modulate IL-12 bioactivity is described for the first time. In an effort to determine the mechanism(s) by which heparin influences IL-12 bioactivity, comprehensive biophysical and cell-based studies were performed. Specifically, we assessed the ability of heparin to: (1) stabilize IL-12 conformation; (2) protect IL-12 from proteolytic degradation; (3) induce oligomerization of IL-12; and (4) enhance IL-12 binding to cell's surfaces and IL-12 receptors. Results of this study are important in that they contribute significantly to our understanding of the immunobiology of IL-12 and potentially other IL-12 family cytokines which have been recently suggested as having more influence on shaping immunity than any other cytokine family^34^.

## Materials and Methods

### IL-12 bioactivity assays

Recombinant hIL-12 was purified from hIL-12-expressing HEK-293 cells as described previously^33^. Low molecular weight heparin, HS, hyaluronic acid, chondroitin sulfate and dextran were purchased from Sigma-Aldrich.

The IL-2-independent, IL-12-responsive human natural killer cell line, NK-92MI (CRL-2408^TM^; ATCC), was cultured in complete media consisting of Alpha MEM supplemented with 12% fetal bovine serum, 12% horse serum, 1% penicillin/streptomycin, 0.2 mM inositol, 0.02 mM folic acid, and 0.1 mM 2-mercaptoethanol. Cell density was maintained between 1 × 10^5^ and 1 × 10^6^ viable cells/mL using a 1:3 split ratio. Peripheral blood mononuclear cells (PBMCs) were isolated from whole blood on a density gradient (Lympholyte H; Cedarlane Labs). Whole blood was either collected from healthy donors, under informed consent, as approved by the Institutional Review Board at the University of Arkansas, or purchased, deidentified, from the New York Blood Center. All experiments were performed in accordance with relevant guidelines and regulations at the University of Arkansas, the University of North Carolina and North Carolina State University.

IFN-γ secretion from NK-92MI cells and PBMCs was used as an indicator of hIL-12 bioactivity. NK-92MI cells and PBMCs were seeded in a 96-well plate at 20,000 and 100,000 cells/well, respectively. hIL-12 was added to achieve final concentrations from 0.04 to 5 ng/ml. Heparin, HS, hyaluronic acid, chondroitin sulfate or dextran was added to a final concentration of 10 µg/ml. Cells in hIL-12 alone or culture media alone served as controls. After 24–48 hours, hIL-12-dependent secretion of IFN-γ into the supernatant of the culture was quantified via enzyme-linked immunosorbent assay (ELISA) (88–7317; eBiosciences). For PBMC subset analysis, stimulated cells were analyzed for intracellular IFN-γ expression on a BD Celesta flow cytometer (BD Biosciences). Additional details are included in Supplementary Methods.

HEK-Blue^TM^ IL-12 cells (Invivogen) express a STAT4-inducible secreted embryonic alkaline phosphatase (SEAP) reporter gene that is triggered upon binding of IL-12 to IL-12R. HEK-Blue^TM^ IL-12 cells were seeded onto a 96 well plate at 50,000 cells/well and exposed to media containing 0.04 to 20 ng/ml hIL-12 with or without 10 µg/ml heparin. After 12 hours, SEAP concentrations in supernatants were detected through the addition of Quanti-Blue^TM^ (Invivogen) and quantified via absorbance readings at 650 nm.

### IL-12-GAG binding studies

Binding affinities between hIL-12 and various GAGs were quantified by isothermal titration calorimetry on an iTC200 (MicroCal Inc.). Both hIL-12 and GAGs were prepared in 10 mM sodium phosphate buffer with 100 mM sodium chloride at pH 7.2. All samples were subjected to high speed centrifugation to remove any suspended particulate material and degassed to remove dissolved air before titration. GAGs (2 mM) were titrated into hIL-12 (200 µM) for a total of 30 injections. Titrations were performed at physiological temperature (37 °C). Titration curves were fit to the one-set of sites binding model using Origin^TM^ v7.0 software to derive binding constants.

### Proteolytic digestion assays

To evaluate hIL-12 degradation in spent media, NK-92MI cells were cultured in media without serum for 3 days at a starting cell density of 1 × 10^6^ cells/ml. Spent media were collected, centrifuged, 0.2 μm-filtered and co-incubated with 500 pg/ml hIL-12 ± 10 μg/ml heparin. Fresh cell culture media with 50 μg/ml bovine serum albumin as a stabilizing protein was used as a negative control. Fresh cell culture media with 0.125% trypsin was used as a positive control. Samples from each treatment were collected at 0, 24, 48 and 72 hours. Full length hIL-12p70 concentrations were quantified via ELISA (88-7126; eBiosciences).

To evaluate hIL-12 degradation by known proteases, trypsin, chymotrypsin, thermolysin, thrombin and protease inhibitor cocktail tablets were purchased from Sigma-Aldrich. A protease cocktail was prepared by mixing trypsin, chymotrypsin, thrombin and thermolysin in PBS. hIL-12 (1 μg/ml) ± heparin (10 μg/ml) was added to the cocktail at protein to protease molar ratio of 10:1. PBS with 0.1% w/v bovine serum albumin as a stabilizing protein was used as a negative control. The mixture was incubated at room temperature and samples were collected at 0, 1, 2, 5, 10, 15, 20 and 30 min. Full length hIL-12p70 concentrations were quantified via ELISA (88-7126; eBiosciences).

### Size exclusion chromatography

Oligomerization of hIL-12 in the presence of heparin was observed on a Sephacryl S-200 size exclusion chromatography column connected to an AKTA fast protein liquid chromatography system. All samples were resolved at room temperature at a flow rate of 1 mL/min in 10 mM sodium phosphate buffer (pH 7.2) containing 100 mM NaCl. The apparent molecular mass of the hIL-12 samples was determined against a standard curve constructed under similar conditions using common protein standards – β-amylase (200 kDa), alcohol dehyrogenase (150 kDa), conalbumin (75 kDa), ovalbumin (45 kDa), carbonic anhydrase (29 kDa) and RNaseA (13.7 kDa).

### Circular Dichroism

The secondary structure of hIL-12, in the presence and absence of heparin, was monitored by far-UV circular dichroism (CD) using a Jasco-710 spectropolarimeter. Samples contained 100 µM hIL-12 in 10 mM sodium phosphate buffer (pH 7.2) with 100 mM sodium chloride. A total of 15 scans were acquired at 25 °C with a scan speed of 50 nm/minute. Data for hIL-12 with heparin at a 1:5 molar ratio was collected under the same conditions. Appropriate blank titrations were performed to eliminate background noise. Processed data was expressed in molar ellipticity (deg. cm^2^. dmol^−1^).

### Differential Scanning Calorimetry

Thermal denaturation of hIL-12 (100 µM), in the absence and presence of heparin, was performed on a NANO DSC III differential scanning calorimeter (TA Instruments) with a ramping temperature of 1 °C/min spanning from 10 °C to 90 °C in 10 M sodium phosphate buffer (pH 7.2) containing 100 mM sodium chloride. Thermodynamic values were calculated using Origin^TM^ version 7.0 software

### Equilibrium unfolding and ANS binding

Guanidine hydrochloride-induced unfolding was performed on 10 µM hIL-12 in the presence and absence of heparin. The equilibrium unfolding data was plotted to derive the melting concentration (C_m_) and ΔG (H_2_O). The surface accessible non-polar surfaces in hIL-12, in the presence and absence of heparin, was monitored by 8-anilinonaphthalene-1-sulfonic acid (ANS) fluorescence. All fluorescence experiments were performed at 25 °C on a Hitachi F2500 spectrofluorimeter. The excitation wavelength was set to 280 nm and bandwidths for excitation and emission were set to 2.5 and 10 nm, respectively. A stock solution of 20 mM ANS solution was used for titration into 10 µM hIL12 in 10 mM sodium phosphate buffer (pH 7.2) containing 100 mM sodium chloride. Samples were excited at 380 nm and emission spectra were monitored between 450 to 600 nm with a peak observed at 500 nm. Data from the ANS binding assay were overlaid to identify differences in surface hydrophobicity.

### Small-angle X-ray Scattering (SAXS) Analysis

SAXS data of hIL-12, in the absence and presence of heparin, was acquired at the Cornell High Energy Synchrotron Source (CHESS) beamline G1 source. Inline size exclusion chromatography (SEC) was used to minimize polydispersity. hIL-12 (150 µM), with and without low molecular weight heparin (in ten-fold excess) was loaded onto a Sephacryl-S 300 column attached to an AKTA explorer (GE Healthcare). Eluted protein sample entered the flow cell of the BioSAXS at CHESS and was subjected to X-ray beam exposure. Beamline characteristics used for acquiring the data were as follows: energy = 9.968 keV(1.257 A); beam diameter = 250 µm × 250 µm; photon flux = 1.6 × 10^11^ photons/sec. The detector used was a dual Pilatus 100K-S with a q-space range between 0.006 to 0.8 Å-1. Data acquired was processed using BioXTAS RAW software for performing the buffer subtraction. Buffer subtracted plots were analyzed using the ATSAS program^35^ with a sequence of steps to obtain an average low resolution structure.

### Heparin binding and IL-12 bioactivity on IL-12R mutant and wild-type cells

Both IL-12 receptor subunits, IL-12Rβ1 and IL-12Rβ2, were functionally deleted from NK-92MI cells via CRISPR/Cas9 genome editing (see Supplementary Methods). The resulting mutants are denoted as IL12Rβ1^mut^/IL12Rβ2^mut^ NK-92MI cells. For analysis of cell surface binding, wild-type NK-92MI cells, IL12Rβ1^mut^/IL12Rβ2^mut^ NK-92MI cells, PBMCs and HEK-293 cells (CRL-1573; ATCC) were incubated at 4 °C, to inhibit cellular uptake, with Alexa Fluor 647 (ThermoScientific)-labeled hIL-12 (AF647-IL12) (10 ng/ml), in the presence and absence of 10 μg/ml heparin. Cells in culture media alone served as controls. After one hour, cells were analyzed on a BD FACSCantoII or a BD Celesta (BD Biosciences).

To assess the effect of IL12R deletion on heparin binding, parental NK-92MI and IL12Rβ1^mut^/IL12Rβ2^mut^ cells were exposed to 10 μg/ml heparin, labeled with Cyanine5 (heparin-Cy5) (Nanocs), for 1 hour at 4 °C. Cells in culture media alone served as controls. After one hour, cells were washed once with cold PBS and analyzed on a BD FACSCantoII.

To assess the effect of heparin on hIL-12 bioactivity in IL12R mutant cells, parental NK-92MI and IL12Rβ1^mut^/IL12Rβ2^mut^ cells were cultured with 200 pg/ml hIL-12 ± 10 μg/ml heparin for 24 hours. IFN-γ production was quantified by ELISA as described above.

### Statistical analysis

All experiments were carried out in triplicate or quadruplicate. Where appropriate, data are presented as means ± standard deviation. Where indicated, analysis of variance (ANOVA) or Student’s t-test were performed using Prism 7 software (GraphPad Software, Inc, La Jolla, CA). Statistical significance was accepted at the p ≤ 0.05 level.

### Data availability statement

All data and relevant materials are available from the corresponding author upon reasonable request.

## Results

### Effect of heparin and other GAGs on hIL-12 bioactivity

**Figure 1:**
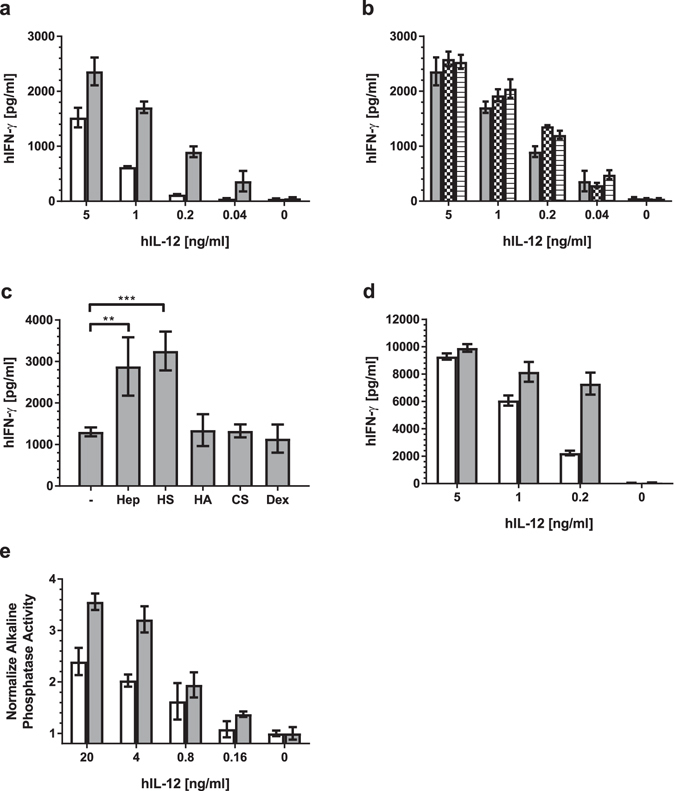
Effect of heparin and other GAGs on hIL-12 bioactivity. (**a**) hIFN-γ production by NK-92MI cells when incubated with media containing hIL-12 alone (white bars) or with 10 µg/ml heparin (gray bars) was quantified using ELISA. Heparin significantly enhanced hIL-12 bioactivity (p < 0.0001 vs. hIL-12 alone via two-way ANOVA) (**b**) hIFN-γ production by NK-92MI cells when heparin (10 µg/ml) was added to NK-92MI cells at the same time as hIL-12 (gray bars), 30 mins prior to hIL-12 (checkered bars) or 30 mins after hIL-12 (horizontal). The order of heparin addition had no significant effect on hIL-12 activity (p > 0.05 for all comparisons within each hIL-12 concentration via two-way ANOVA with Tukey’s post hoc correction). (**c**) hIFN-γ production by NK-92MI cells cultured in media containing 200 ng/ml hIL-12 alone (−) or with heparin (Hep), heparin sulfate (HS), hyaluronic acid (HA), chondroitin sulfate (CS), or dextran (Dex) at 10 µg/ml was quantified using ELISA. Asterisks indicate a significant difference between indicated groups (*=p < 0.01 and ***=p < 0.001 via one-way ANOVA with Dunnett’s post hoc correction). (**d**) hIFN-γ production by PBMCs when incubated with media containing hIL-12 alone (white bars) or with 10 µg/ml heparin (gray bars) was quantified using ELISA. Heparin significantly enhanced hIL-12 bioactivity (p < 0.0001 vs. hIL-12 alone via two-way ANOVA). (**e**) Alkaline phosphate activity by HEK-Blue™ IL-12 cells in response to incubation with media containing hIL-12 alone (white bars) or with 10 µg/ml heparin (gray bars) was determined in accordance with manufacturer’s instructions. Heparin significantly enhanced hIL-12 bioactivity (p < 0.0001 vs. hIL-12 alone via two-way ANOVA). All data are represented as mean ± standard deviation from triplicate samples. All experiments were repeated at least twice with similar results.

IFN-γ production from NK-92MI cells exposed to hIL-12 in the presence and absence of heparin was used as a measure of hIL-12 bioactivity. NK-92MI cells produced low levels of IFN-γ (47.3 ± 7.0 pg/ml) in the absence of hIL-12 (Fig. 1a). Upon exposure to 0.04 to 5 ng/ml hIL-12, IFN-γ concentrations increased steadily from 48 ± 7 to 1523 ± 178 pg/ml. Overall, the addition of heparin significantly augmented hIL-12-induced IFN-γ production by 55 to 661% (p < 0.05 vs. hIL-12 alone via ANOVA). Treatment of cells with heparin alone (0 pg/ml hIL-12) had no effect on IFN-γ production (p > 0.05 vs. untreated controls via t-test).

To determine if the order of heparin addition influenced hIL-12 bioactivity, heparin was added to culture medium 30 minutes before hIL-12, at the same time as hIL-12 or 30 minutes after hIL-12. The order of heparin addition was found to have no effect (p > 0.05 vs. hIL-12 alone via ANOVA) (Fig. 1b).

The effect(s) of other common GAGs, including HS, hyaluronic acid, and chondroitin sulfate, on hIL-12 bioactivity were also examined. Similar to heparin, HS enhanced hIL-12-mediated production of IFN-γ by about 2.5 times (Fig. 1c). Conversely, chondroitin sulfate, hyaluronic acid and dextran, as a non-GAG control polysaccharide, had no discernable effect on hIL-12 bioactivity.

To determine if the heparin-induced increase in hIL-12 bioactivity was a widespread phenomenon, similar experiments were performed in human PBMCs and HEK-Blue^TM^ IL-12 sensing cells. Indeed, heparin significantly enhanced IL-12 bioactivity on both human PBMCs (Fig. 1d) and HEK-Blue^TM^ IL-12 cells (Fig. 1e). In PBMCs, the effect was most pronounced at 200 ng/ml hIL-12 where IFN-γ production was increased by more than 3-fold in the presence of heparin. The enhancing properties of heparin decreased at higher hIL-12 concentrations. An analysis of intracellular IFN-γ expression by PBMC subsets via flow cytometry revealed that NK cells, and not CD4^+^ or CD8^+^ T cells, were primarily responsible for the observed increase in IFN-γ production (Supplementary Fig. S1).

### Effect of heparin and other GAGs on hIL-12 binding

ITC experiments were performed to compare the binding affinities between several GAGs and hIL-12. The affinity of hIL-12 to heparin was found to be in the low micromolar range with an apparent K_d_ value of ~23 µM (Table 1). HS also bound to hIL-12 in the low micromolar range (K_d_ ~9 µM). However, no binding was observed between hIL-12 and the other GAGs or dextran examined. Representative isothermograms are shown in Supplementary Fig. S2.

**Table 1:** Binding parameters of various GAGs interacting with IL-12 derived from ITC analysis.

	K_d_ (µM)	Stoichiometry/number of binding sites (n)	ΔH [cal/mol]	TΔS [cal/mol/°K]	ΔG [cal/mol/°K]
Heparin	23 ± 3.1	0.98 ± 0.02	−2526.2	4350.8	−1824.6
Heparan sulfate (HS)	9 ± 1.2	1.01 ± 0.02	−1920.2	4350.8	−2430.6
Chondroitin sulfate (CS)	ND	ND	ND	ND	ND
Hyaluronic Acid (HA)	ND	ND	ND	ND	ND
Dextran	ND	ND	ND	ND	ND

*ND - not determined.

The stoichiometry of binding, obtained from fitting the ITC data to a one-set of sites binding model, was approximately 1 for both HS/IL12 and heparin/IL-12 complexes (Table 1). This indicates a single binding site for heparin/HS on hIL-12. Heparin/HS binding was accompanied by a large decrease in enthalpy (ΔH) and an increase in entropy (ΔS). These changes indicate that the IL-12/heparin binding is facilitated by both hydrophobic and electrostatic interactions.

### Effect of heparin in preventing proteolytic degradation of hIL-12

**Figure 2:**
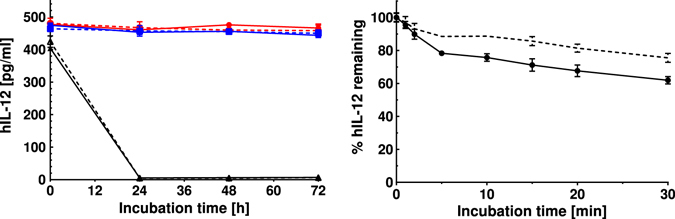
Effect of heparin on proteolytic degradation of hIL-12. (**a**) hIL-12 degradation when incubated with (open symbols) and without heparin (closed symbols) in spent media (squares), fresh AMEM containing BSA (circles) or fresh AMEM containing 0.125% trypsin (triangles). Samples were collected at intervals of 0, 24, 48 and 72 hours and hIL-12 concentrations were quantified via ELISA. Heparin did not affect hIL-12 degradation (p > 0.05 vs. hIL-12 alone via two-way ANOVA). (**b**) hIL-12 degradation when incubated with (open circles) and without heparin (filled circles) in protease cocktail containing trypsin, chymotrypsin, thrombin and thermolysin. Proteases were added to hIL-12 solution at a protein to protease molar ratio of 10:1. Samples were collected and neutralized with protease inhibitor cocktail at intervals of 0, 1, 2, 5, 10, 15, 20 and 30 min and hIL-12 concentrations were quantified via ELISA. Heparin significantly inhibited hIL-12 degradation by protease cocktails. Asterisks indicate a significant difference between groups at a particular time point (*=p < 0.01 vs. hIL-12 alone via t-test). All data are represented as mean ± standard deviation from triplicate samples. All experiments were repeated at least twice with similar results.

To determine if the heparin-dependent increase in hIL-12 bioactivity could be attributed to protection from proteolytic enzymes in cell culture media, the time-dependent degradation of hIL-12 in fresh and spent media, with and without heparin, was quantified. Spent media were obtained from NK-92MI cells cultured at high density for 24 hours without serum supplementation. After up to 72 hours of incubation, there was no appreciable degradation of hIL-12 in spent media regardless of heparin addition (Fig. 2a). In fact, concentrations of hIL-12 in spent media or fresh media were indistinguishable. The addition of a strong proteolytic enzyme, trypsin, completely degraded hIL-12, apparently irrespective of heparin addition, within 24 hours. In a follow up experiment, hIL-12 alone or hIL-12 with heparin, was cultured with a protease cocktail for up to 30 minutes (Fig. 2b). At this shorter time scale, it became apparent that heparin partially protects hIL-12 from strong proteolytic enzymes.

### Analysis of heparin-hIL-12 interactions

Following up on the ITC binding studies, the nature of the interactions between heparin and hIL-12 was characterized by SEC and SAXS. SEC revealed that hIL-12 migrated with an apparent molecular mass of 103.6 kDa (Table 2). The difference between this apparent molecular mass from SEC and the experimental molecular mass of hIL-12 (65 kDa) obtained from our previous ESI-MS analysis^33^, could be attributed to the extensive glycosylation pattern that is found in this class of cytokines^36–40^ and the extended, non-globular structure of hIL-12^33, 41^. In the presence of heparin, hIL-12 showed an apparent molecular mass of ~172.9 kDa suggesting that binding of heparin likely induces dimerization of the protein. The marginal difference between the expected molecular mass and the observed molecular mass, calculated from the SEC experiment, can be attributed to the non-globular nature of the IL12-heparin complex and also the minor uncertainties introduced due to data extrapolation on the standard molecular mass curve which did not include proteins of molecular mass greater than 180 kDa.

**Table 2:** Comparison of MW_app_ of hIL-12 in the absence and presence of heparin using different methods.

	ESI-MS	GPC Sephacryl-S200	SEC-SAXS
−Heparin	65 kDa^33^	~100 kDa	~70 kDa
+Heparin	Not obtained	~170 kDa	~200 kDa

SAXS analysis indicated that the average molecular mass of hIL-12, calculated from the radius of gyration, was approximately 70 kDa (Table 2). This is in good agreement with the predicted molecular mass of hIL-12 including glycosylation. For heparin-hIL-12 complexes, the average molecular mass was approximately ~200 kDa which corroborates the SEC data.

### Effect of heparin on hIL-12 conformation and stability

**Figure 3:**
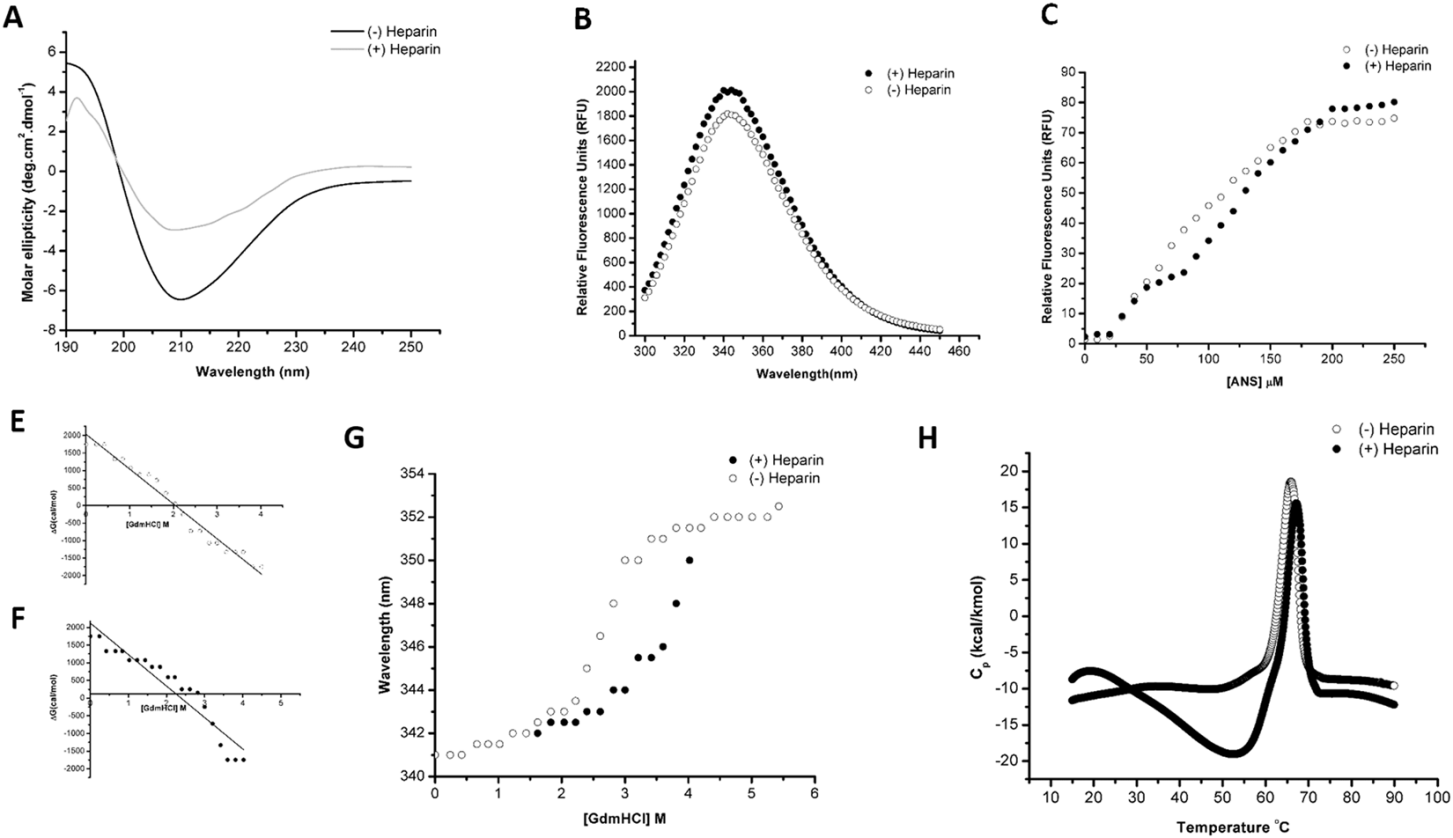
Effect of heparin on the conformation and stability of hIL12. (**A**) Overlay of the far-UV CD spectra (190 nm–250 nm) of hIL-12, in the absence (solid black) and presence (solid gray) of heparin. (**B**) Overlay of the steady-state fluorescence emission spectra on hIL-12 in the absence (empty circle) and presence (filled circle) of heparin. (**C**) Overlay of the equilibrium unfolding curves of IL-12, in the presence (filled circle) and absence (empty circle) of heparin. (**D**,**E**). A plot of the concentration of GdmCl [M] versus ΔG (cal/mol) to determine the concentration (C_m_) of the denaturant required for denaturation of 50% of the protein population, present in the native conformation. (**G**) Overlay of the ANS binding curves of hIL-12 in the absence (empty circle) and presence (filled circle) of heparin. (**H**) Overlay of the thermal denaturation curves of hIL12 in the absence (empty circle) and presence (filled circle) of heparin. Thermal denaturation was carried out by ramping the temperature at a rate of 1 °C/min. Data obtained was plotted as heat capacity at constant pressure (Cp) versus temperature (°C) to obtain the T_m_ of hIL12.

Far-UV CD measurements were performed to determine if heparin influenced the secondary structure of hIL-12. In the absence of heparin, hIL-12 showed a strong negative ellipticity band at around 210 nm (Fig. 3a) and a shoulder around 222 nm indicating the presence of alpha-helix and beta-sheet secondary structures. Incubating hIL-12 with heparin revealed only minor changes in the shape of the spectrum. These results suggest that heparin does not induce discernable backbone conformational changes in IL-12.

Intrinsic tryptophan fluorescence is a sensitive technique to monitor subtle tertiary structural changes induced by ligand binding. An overlay of intrinsic fluorescence emission spectra in the absence and presence of heparin for hIL12 showed an identical emission maxima at 341 nm (Fig. 3B) suggesting there was no significant change in the microenvironment of tryptophan upon binding to heparin. A marginal increase in the relative fluorescence intensity in the presence of heparin could be attributed to an increased rigidity in the tertiary structure of the protein with an enhanced hydrophobic content around the sites of tryptophan.

Similar to thermal unfolding, proteins undergo denaturation in the presence of strong chaotropes such as, guanidinium salts or urea. In this study, hIL-12 was subjected to guanidinium chloride-based equilibrium unfolding. It was observed that hIL-12 is moderately stabilized in the presence of heparin as evidenced by an increase in the C_m_ value [concentration of the denaturant at which 50% of the protein population is in the denatured state(s)] from 2.0 M to 2.5 M (Fig. 3C–E).

ANS binding assay was used to monitor surface hydrophobicity and solvent accessibility of surface residues in hIL-12 in the absence and presence of heparin. ANS is a hydrophobic fluorescent dye which binds to solvent-exposed non-polar surface of proteins. Emission spectrum of ANS upon binding to IL-12 shows an emission maximum at 500 nm. The ANS concentration-dependent binding curve (Fig. 3F) shows a steady increase in the emission intensity at 500 nm (Fig. 3F) in the range of 0 – 200 μM and plateaus at higher concentrations. The ANS concentration-dependent binding curve in the presence of heparin shows a similar trend. However, the 500 nm emission intensity, in the presence of heparin, is marginally lower in the ANS concentration of 75 μM – 200 μM suggesting that the binding of heparin only causes a subtle conformational change in IL-12.

To understand the effect of heparin on the thermal stability of hIL-12, protein unfolding was monitored using differential scanning calorimetry. hIL-12 in the absence of heparin, showed a melting temperature (T_m_) of ~ 65 °C (Fig. 3G). The unfolding process was irreversible as the cooling cycle did not show a sharp peak as observed during the heating cycle. In the presence of heparin, T_m_ increased marginally by only 1.2 °C.

### Effect of heparin on cell surface binding of hIL-12

**Figure 4:**
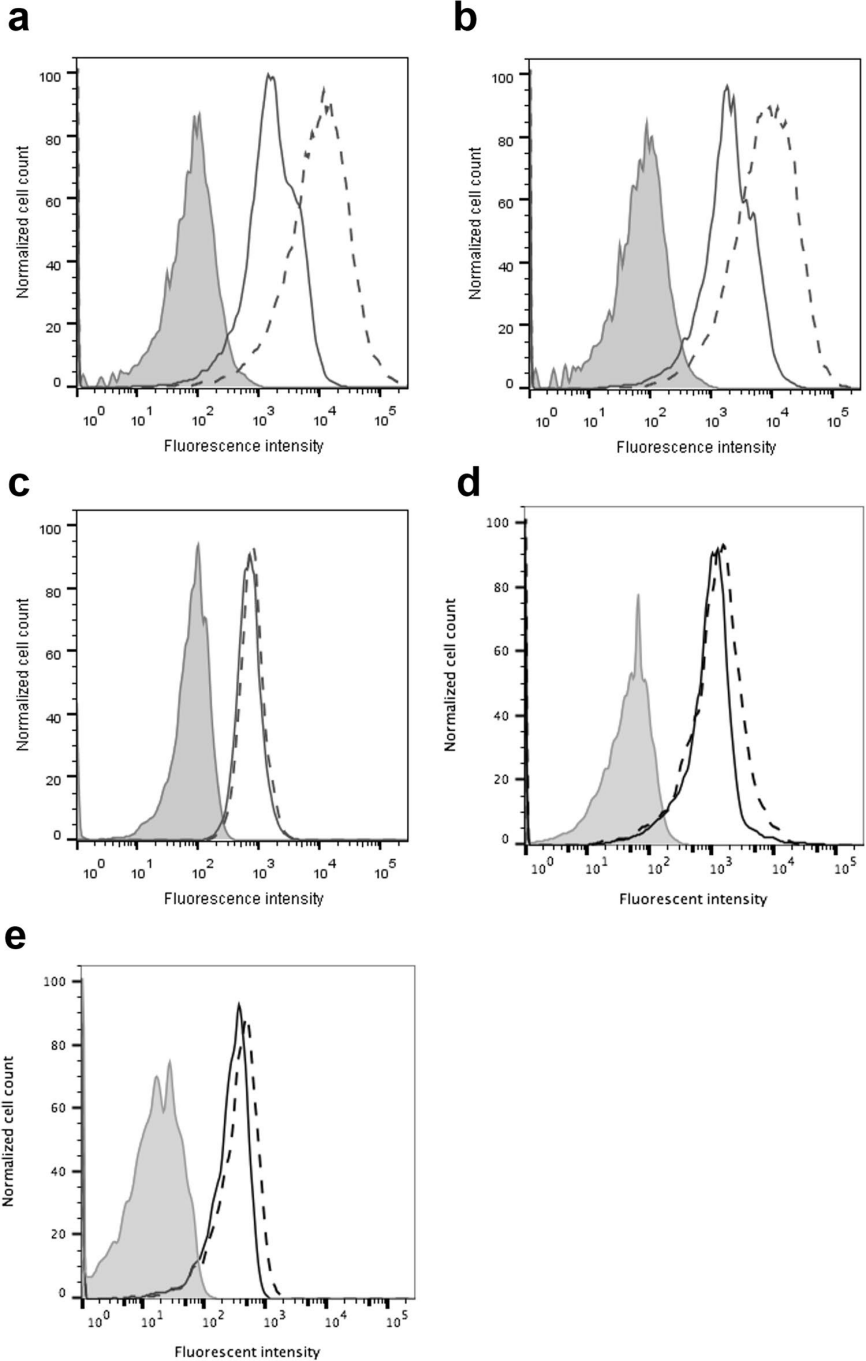
Effect of heparin on hIL-12 binding to cell surfaces. (**a**) NK-92MI cells, (**b**) IL-12Rβ1^mut^/IL-12Rβ2^mut^ NK-92MI cells, (**d**) HEK-293 cells or (**e**) PBMCs cultured with AF647-IL12 alone (solid line) or AF647-IL12 plus 10 μg/ml heparin (dashed line). Untreated cells (filled histogram) served as a negative control. (**c**) NK-92MI cells (solid line) and IL-12Rβ1^mut^/IL-12Rβ2^mut^ NK-92MI cells (dashed line) were cultured for 1 hour at 4 °C with heparin-Cy5. Untreated NK-92MI cells (filled histogram) served as a negative control. Each panel shows the relative amounts of AF647-IL12 or heparin-Cy5 binding to cells as assessed via flow cytometry.

Binding of AF647-hIL12 to NK-92MI cells, PBMCs and HEK cells in the presence and absence of heparin was assessed via flow cytometry. The addition of AF647-hIL12 alone to NK-92MI cells resulted in a significant shift in cell surface fluorescence compared to unstained controls (Fig. 4a). When exogenous heparin was included, the mean fluorescence intensity due to AF647-hIL12 binding increased from 2,200 to 12,561.

Recognizing that heparin could be facilitating non-specific binding to the cell surface, i.e. binding to moieties other than the IL-12 receptors, the experiment was repeated in NK-92MI cells in which both IL-12 receptor subunits, IL12Rβ1 and IL12Rβ2, were functionally deleted via CRISPR-Cas9 gene editing (see Supplementary Methods). Indeed, disrupting the heterodimeric IL-12 receptor had no effect on the increase in AF647-hIL12 binding to the cell surface in the presence of heparin (Fig. 4b). Additionally, heparin binding, as visualized by heparin-Cy5, was not influenced by the IL12R deletion (Fig. 4c).

Follow up western blot and flow cytometry assays, revealed that the IL12Rβ1^mut^/IL12Rβ2^mut^ NK-92MI cells still expressed some portion of the IL12R subunits (Supplementary Fig. S3). Therefore, an additional experiment was performed to measure hIL-12 binding to HEK-293 cells which do not express IL12R and PBMCs which mostly do not express IL12R. Figure 4d and e demonstrated that heparin modestly increases IL-12 binding to the surfaces of HEK-293 cells and PBMCs.

**Figure 5:**
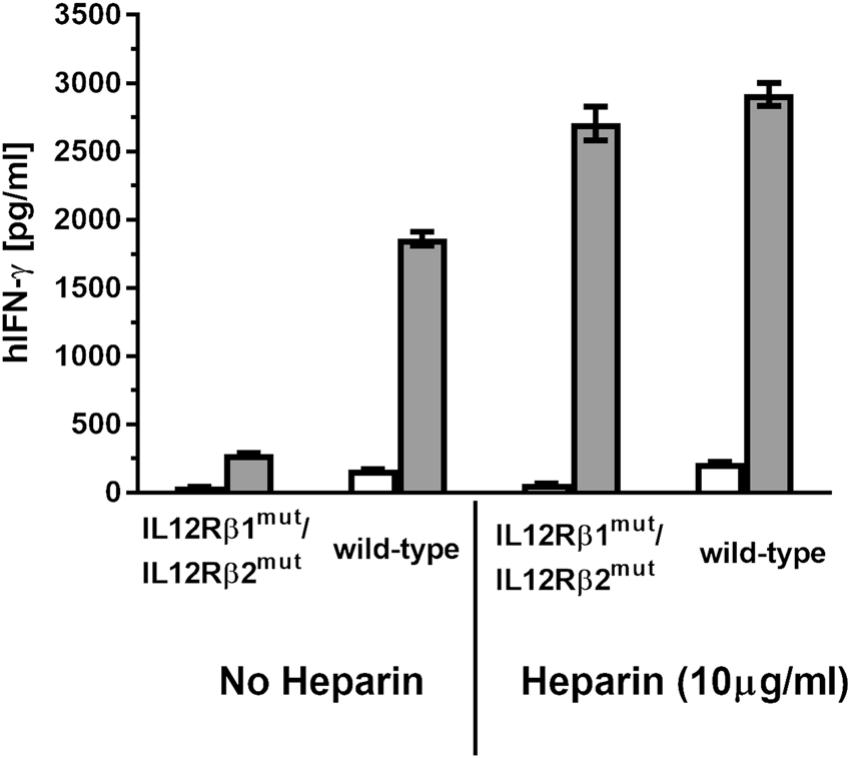
Heparin-induced recovery of hIL-12 activity. hIFN-γ production by parental NK-92MI cells and IL-12Rβ1^mut^/IL-12Rβ2^mut^ NK-92MI cells treated with 0 ng/ml hIL-12 (white bars) or 200 ng/ml hIL-12 (gray bars) in the presence and absence of 10 ng/ml heparin was quantified via ELISA. All data are represented as mean ± standard deviation from triplicate samples.

Perhaps most interestingly, even though hIL-12 alone is not functional in IL12Rβ1^mut^/IL12Rβ2^mut^ NK-92MI cells, the addition of heparin almost completely restored hIL-12 bioactivity (Fig. 5). In fact, IFN-γ production by the IL-12R mutant cells treated with hIL-12 and heparin was greater than 90% of the IFN-γ production by wild-type NK-92MI cells treated with hIL-12 plus heparin.

## Discussion

Our previous study demonstrated that hIL-12 binds to heparin specifically and that heparin binding sites are primarily located on the p40 subunit of hIL-12^33^. This study is the first to unambiguously demonstrate that heparin positively modulates the activity of hIL-12 (Fig. 1). Initial bioactivity studies were performed on a human NK cell line and subsequently confirmed using human PBMCs and an IL-12-sensing HEK cell line. Thus, the heparin-induced increase in hIL-12 activity appears to be widespread. A PBMC subset analysis revealed that NK cells, and not CD4^+^ nor CD8^+^ T cells, were responsible for the increase in IFN-γ production in response to stimulation with hIL-12 and heparin (Supplemental Fig. S1).

Binding and modulation of hIL-12 activity was restricted to heparin and HS, the two most N-sulfated GAGs. Chondroitin sulfate and hyaluronic acid did not bind to hIL-12 nor did they have an effect on hIL-12 bioactivity. The fact that dextran, chondroitin sulfate and hyaluronic acid did not influence hIL-12 bioactivity indicates that a high density of sulfation is critical for binding and modulating hIL-12.

ITC binding analyses demonstrated that hIL-12 contains a distinct, accessible heparin binding pocket. The low micromolar binding affinities for heparin and HS, together with moderate increases in entropy and decreases in enthalpy are indicative of a robust, specific electrostatic and non-polar interaction.

SEC and SAXS data demonstrated that hIL-12 predominantly forms dimeric structures in the presence of heparin (Table 2). This dimerization/oligomerization could help stabilize the IL-12/receptor complex. Similar observations have been made with other interleukins. The extracellular domain of IL-5Rα binds to a homodimeric form of IL-5 attaining a critical “wrench-like” structural conformation^42^. Likewise, IL-22 was shown to form dimers and tetramers in solution and organize into a V-shaped dimeric conformation binding to IL-22R1 receptor^43^. The effect of heparin on stabilizing the IL-12/receptor complex is the subject of ongoing studies.

With respect to other mechanism(s) by which heparin modulates hIL-12 activity, we explored a number of possibilities based on the heparin literature. Heparin has been shown to protect proteins, such as bFGF, from the hydrolytic action of plasmin and other proteases present in the extracellular milieu^44^. However, our data showed that hIL-12 was not significantly degraded by proteases or any other molecules that may be present in spent media (Fig. 2a). As expected, trypsin, used as a positive control, quickly and completely degraded hIL-12 equally in the presence and absence of heparin. Because trypsin digested all of the hIL-12 within the first time point, i.e. 24 hours, a subsequent experiment with a defined protease cocktail was performed over a shorter time frame. Within 30 minutes, heparin was found to reduce protease-driven hIL-12 degradation by up to 14% (Fig. 2b). However, it should be noted that all hIL-12, regardless of heparin inclusion, is expected to be inactivated by the protease cocktail within a few hours. Thus, although heparin can somewhat protect hIL-12 from strong proteolytic degradation, this is not a plausible mechanism for the increased hIL-12 activity *in vitro* that we observed.

Conformational analysis of hIL-12 in the presence and absence of heparin was performed as other heparin-binding proteins have been shown to be stabilized with heparin. Biophysical characterization of heparin-hIL-12 binding via far-UV CD measurements, folding studies, ANS binding and thermal stability assays demonstrate that heparin causes a slight change in hIL-12 conformation but no gross secondary or tertiary structural changes (Fig. 3). There is also a minor increase in thermal stability. Taken together, conformational and stability changes are not so significant to suggest that they would lead to a major shift in hIL-12 bioactivity.

The strongest evidence for the mechanism by which heparin enhances hIL-12 bioactivity points to heparin serving as a co-receptor for hIL-12. Heparin clearly increases the concentration of hIL-12 at cell surfaces (Fig. 4). The effect was more pronounced in cells expressing IL-12R, e.g. mutant and wild-type NK-92MI. Nevertheless a small increase in hIL-12 on the cell surface could also be found in cells expressing relatively little (PBMCs) or no IL-12R (HEK-293). That heparin bind to cell surfaces at high levels suggest that exogenous heparin is capable of maintaining a reservoir of IL-12 at the cell surface. Heparin may also control the kinetics of association and dissociation of IL-12 to IL-12Rβ1 and IL-12Rβ2. Ibrahami *et al*.^45^, studying the kinetics of the FGF signaling complex assembly, suggested that heparin regulates the cell proliferation activity due to FGF by switching FGFR between high and low affinity states. While heparin may specifically bind to IL-12R just as it does to FGFR, follow up studies showed that heparin binds equally to wild type and IL12Rβ1^mut^/IL12Rβ2^mut^ cells (Fig. 4c). Thus, it appears that heparin is binding at high levels to numerous proteins on the cell surface.

Most surprisingly, heparin was shown to facilitate hIL-12 signaling in NK-92MI cells in which both IL-12Rβ1 and IL-12Rβ2 had been functionally deleted (Fig. 5). A follow up analysis of IL12R expression via flow cytometry and western blot indicate that forms of both IL-12Rβ1 and IL-12Rβ2, although non-functional, were still expressed by the CRISPR/Cas9-modified NK-92MI cells (Supplementary Fig. S3). Thus, it is likely that during the Cas9 dsDNA break, random insertion/deletion of nucleotides resulted in nonsynonomous mutations which allowed for expression of mutant IL12R subunits. Experiments to sequence the mutant IL12R subunits are planned.

In the absence of heparin, hIL-12 likely has poor affinity to mutated IL-12R and thus loses its ability to signal. In the presence of heparin, the interaction of hIL-12 with IL12Rβ1^mut^ and IL12Rβ2^mut^ appears to be stabilized enough to recover hIL-12 signaling. Using a string tool algorithm developed by our group^46^, we searched the amino acid sequences of IL12Rβ1 and IL12Rβ2 for putative heparin binding segments, e.g. XBXXBX, XBBXBX, BXBBXB, etc. where B is a basic residue and X is a non-basic residue. Results from this search indicated that the extracellular domain (ECD) of IL-12Rβ1 contains 2 very prominent heparin binding segments located at amino acids 293–298 and 454–459. The ECD of IL-12Rβ2 contains 3 potential heparin binding segments, amino acids 53–58, 273–278 and 353–358. Thus, heparin may simultaneously bind to and stabilize hIL-12 with one or both IL-12R subunits. Quantitation of heparin’s influence on the affinities of hIL-12 for wild-type and mutated IL-12R ECDs is the subject of ongoing research.

Data from these studies clearly demonstrate a physiological role for heparin in IL-12 immunobiology. The observed increase in hIL-12 activity in the presence of heparin is rare among interleukins, for which heparin-binding is typically more inhibitory than augmentative. A potential evolutionary explanation for heparin binding is to compartmentalize IL-12 at specific sites. IL-12, when administered systemically, is known to induce life-threatening adverse events^47^. Thus, heparin’s ability to localize IL-12, for example at a site of infection, while increasing its activity may provide a way to limit the systemic toxicity of this potent cytokine. A similar compartmentalization phenomenon has been observed with other cytokines whose systemic dissemination is detrimental^48^. Furthermore, the finding that heparin can recover IL-12 signaling in cells with mutated IL-12R has significant implications for patients with atopic diseases driven by IL-12 family receptor point mutations^49, 50^. These patients are susceptible to mycobacterial infections due to defects in the IL-12/IFN-γ axis. Strategies to use heparin to co-deliver IL-12 are now being developed by our group.

Lastly, given the similarities among cytokines in the IL-12 family, our data also imply a role for heparin in modulating the activities of IL-23, IL-27 and IL-35. IL-23, like IL-12, contains the heparin-binding p40 subunit, while IL-27 and IL-35 contain the Epstein-Barr virus induced gene 3 (EBI3) subunit which is homologous to p40^34, 51^. Because the IL-12 family is central to human immunoregulation^34^, the above studies should motivate further study of heparin as a surprisingly important immunomodulatory agent.

## Electronic supplementary material

Supplementary Methods

Supplementary Figures
