# Supplementary material for: Modulation of Interleukin-12 activity in the presence of heparin
Source: Sci Rep. 2017 Jul 13;7:5360. doi: 10.1038/s41598-017-05382-1 (PMC5509706; doi:10.1038/s41598-017-05382-1)
Supplement: Supplementary file 1 — Supplementary Methods [file 41598_2017_5382_MOESM1_ESM.pdf]

Supplementary Methods for:
Modulation of Interleukin-12 activity in the presence of heparin

Srinivas Jayanthi[1#], Bhanu prasanth Koppolu[2,3#], Khue G. Nguyen[4,5#], Sean G. Smith[2,3], Barbara K. Felber[6], Thallapuranam Krishnaswamy Suresh Kumar[1,4*], David A. Zaharoff[2,3,4,5*]

[1] Department of Chemistry and Biochemistry, University of Arkansas, Fayetteville, AR
[2] Department of Biomedical Engineering, University of Arkansas, Fayetteville, AR
[3] Joint Department of Biomedical Engineering, North Carolina State University and the University of North Carolina-Chapel Hill, Raleigh, NC
[4] Cell and Molecular Biology Program, University of Arkansas, Fayetteville, AR
[5] Department of Microbiology and Immunology, University of North Carolina, Chapel Hill, NC
[6] Human Retrovirus Pathogenesis Section, Vaccine Branch-National Cancer Institute, Frederick, MD

[#] Authors contributed equally

Supplementary Methods

CRISPR/Cas9-mediated deletion of IL12Rβ1 and IL12Rβ2

Two 20-bp guide sequences, (5'-GGAGCACTCGTAACGATCAC -3') and (5'-CAGGATTAACTTGTTACGTC-3'), targeting DNA within exon 305 – 419 of *IL12Rβ1* and exon 717 – 1004 of *IL12Rβ2*, respectively, were selected from the CRISPR design tool provided by the Zhang lab at MIT (http://crispr.mit.edu/). Guide sequences were cloned into separate pCas-Guide-EF1a-GFP plasmids via Origene's cloning service. Plasmids were amplified in E. coli and isolated via QIAGEN Plasmid Maxi Kit.

For transfection, NK-92MI cells ($2x10^6$ cells) were re-suspended in 100μl of electroporation buffer (Harvard Apparatus) and added to electroporation cuvettes. Plasmid encoding gRNA (10μg) was added to the electroporation cuvette and mixed well by pipetting. The cell suspension was electroporated at 250 volts, 25 ohms, and 750 μF in a 2 mm cuvette with a ECM630 electroporation system (Harvard Apparatus). The contents of the cuvette were transferred to one well of a 6-well plate containing 1 ml of pre-warmed media. Cells were cultured for 72 hours and sorted for GFP expression on a FACSAriaIII system (BD Biosciences). Sorted cells were subsequently cloned via limiting dilution.

To create a double mutant IL12Rβ1^{mut}/IL12Rβ2^{mut} NK-92MI cell line, IL12Rβ2 was first functionally deleted. An IL-12Rβ2^{mut} clone was selected and propagated for one month to allow transient GFP expression to subside. IL12Rβ1 was subsequently functionally deleted using the transfection method described above. Cells were once again sorted and cloned. Functional deletion of IL12Rβ1 and IL12Rβ2 was verified by loss of IL-12 responsiveness (see Fig. 5).

Analysis of Intracellular IFN-γ expression in PBMC subsets

One million freshly isolate.ed human PBMCs were stimulated with IL-12 alone (200 pg/ml) or IL-12 + heparin (10 μg/ml) for 12 hours in a 96 well plate. Monensin (554724; BD Biosciences) was added for the last 10 hours of stimulation to block intracellular transport. After 12 hours of stimulation, cells were rinsed and stained with viability markers (Fixable Viability Stain 510; BD Biosciences) followed by blocking Fcγ receptors (564220; BD Biosciences) and staining for the following cell surface markers: CD3 (clone: UCHT1), CD19 (clone: SJ25C1), CD4 (clone: RPA-T4), CD8 (clone: RPA-T8), CD56 (clone: B159) and CD45 (clone: HI30). After surface staining, cells were fixed and permeabilized (554715; BD Biosciences) prior to staining for IFN-γ (clone: B27). Twenty thousand live PBMCs were acquired on a BD FACSCelesta (BD Biosciences) and analyzed using FlowJo v10 software.

IL12R expression in mutant NK-92MI via flow cytometry and western blot.

For analysis of IL12R expression via flow cytometry, IL12Rβ1^{mut}/IL12Rβ2^{mut} and wild-type NK-92MI cells were stained with 1 μg/10^6 cells of APC-conjugated anti-IL12Rβ1 FAB839A; R&D Systems), APC-conjugated anti-IL12Rβ2 (FAB1959A; R&D Systems) or APC-conjugates mouse IgG1, k isotype control (550854; BD Biosciences). Cells were rinsed twice in cold PBS and read on a BD FACSCelesta. Data were analyzed using FlowJo v10 software.

For qualitative assessment of IL12R expression via western blot, IL12Rβ1^{mut}/IL12Rβ2^{mut} and wild-type NK-92MI cells as well as HEK-293 cells (negative control) were lysed using RIPA lysis buffer with a protease and phosphatase inhibitor cocktail (78440; ThermoFisher). Homogenates were incubated for 5 minutes and then centrifuged at 13,000 × g for 10 minutes at

4 °C. Cell lysates were collected by withdrawing the supernatant without disturbing the pellet. Protein concentration was estimated via bicinchoninic acid assay (BCA) (23250; Pierce). 50 μg/lane of protein was separated on 12% SDS-PAGE under reduced condition. The proteins were transferred onto a polyvinylidene difluoride (PVDF) membrane and blocked with Odyssey Blocking Buffer (LI-COR Biosciences) at room temperature for 2 h. The membranes were then probed overnight with rabbit anti-IL12Rβ1 antibody (ab96517; Abcam), mouse anti-IL12Rβ2 (MAB19591; R&D Systems) and mouse anti-beta-actin (ab8226; Abcam) in Odyssey Blocking Buffer. Beta-actin was used as the gel loading control. Primary antibodies were used at a dilution of 1:5000. Secondary antibodies, goat anti-rabbit IgG H&L-alkaline phosphatase (ab97048; Abcam) and goat anti-mouse IgG H&L - alkaline phosphatase (ab97020; Abcam) were used at a dilution of 1:5000. After blotting, protein bands were developed with NBT/BCIP substrate solution (34042; ThermoFisher). Qualitative images of blots were taken with an 8.0 megapixel digital camera.
