# Supplementary material for: Modulation of Interleukin-12 activity in the presence of heparin
Source: Sci Rep. 2017 Jul 13;7:5360. doi: 10.1038/s41598-017-05382-1 (PMC5509706; doi:10.1038/s41598-017-05382-1)
Supplement: Supplementary file 2 — Supplementary Figures [file 41598_2017_5382_MOESM2_ESM.pdf]

Supplementary Figures for:
Modulation of Interleukin-12 activity in the presence of heparin

Srinivas Jayanthi[1#], Bhanu prasanth Koppolu[2,3#], Khue G. Nguyen[4,5#], Sean G. Smith[2,3], Barbara K. Felber[6], Thallapuranam Krishnaswamy Suresh Kumar[1,4*], David A. Zaharoff[2,3,4,5*]

[1] Department of Chemistry and Biochemistry, University of Arkansas, Fayetteville, AR
[2] Department of Biomedical Engineering, University of Arkansas, Fayetteville, AR
[3] Joint Department of Biomedical Engineering, North Carolina State University and the University of North Carolina-Chapel Hill, Raleigh, NC
[4] Cell and Molecular Biology Program, University of Arkansas, Fayetteville, AR
[5] Department of Microbiology and Immunology, University of North Carolina, Chapel Hill, NC
[6] Human Retrovirus Pathogenesis Section, Vaccine Branch-National Cancer Institute, Frederick, MD

[#] Authors contributed equally

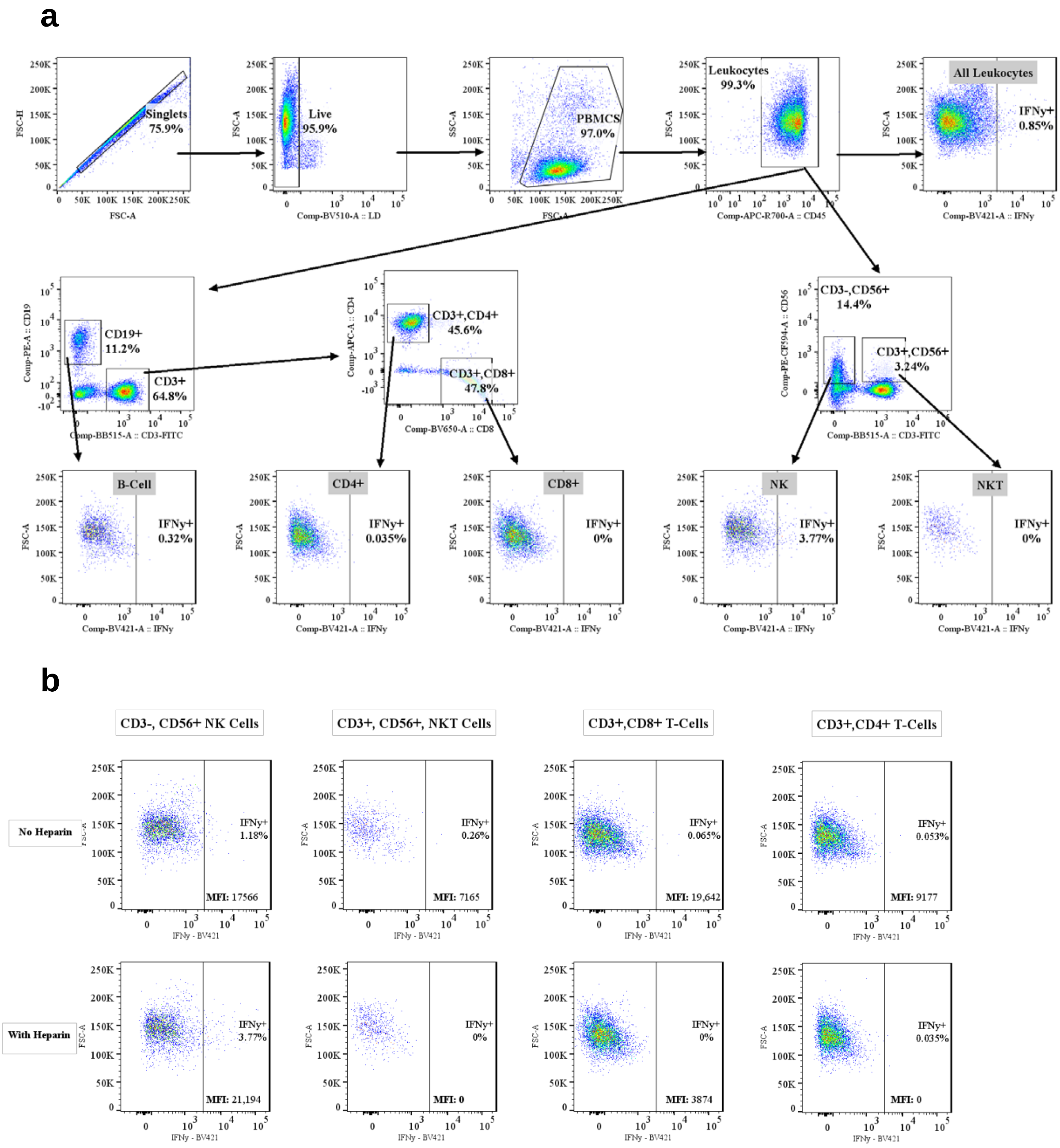
a
Singlets
75.9%
Live
95.9%
PBMCS
97.0%
Leukocytes
99.3%
All Leukocytes
IFNy+
0.85%
FSC-H
FSC-A
SSC-A
Comp-BV510-A :: LD
Comp-APC-R700-A :: CD45
Comp-BV421-A :: IFNy
CD19+
11.2%
CD3+
64.8%
Comp-PE-A :: CD19
Comp-BB515-A :: CD3-FITC
CD3+,CD4+
45.6%
CD3+,CD8+
47.8%
Comp-APC-A :: CD4
Comp-BV650-A :: CD8
CD3-,CD56+
14.4%
CD3+,CD56+
3.24%
Comp-PE-CF594-A :: CD56
B-Cell
IFNy+
0.32%
CD4+
IFNy+
0.035%
CD8+
IFNy+
0%
NK
IFNy+
3.77%
NKT
IFNy+
0%
b
CD3-, CD56+ NK Cells
CD3+, CD56+, NKT Cells
CD3+,CD8+ T-Cells
CD3+,CD4+ T-Cells
No Heparin
IFNy+
1.18%
MFI: 17566
IFNy+
0.26%
MFI: 7165
IFNy+
0.065%
MFI: 19,642
IFNy+
0.053%
MFI: 9177
With Heparin
IFNy+
3.77%
MFI: 21,194
IFNy+
0%
MFI: 0
IFNy+
0%
MFI: 3874
IFNy+
0.035%
MFI: 0
IFNy - BV421

Fig. S1. Intracellular IFN-γ expression among PBMC subsets. Freshly isolated human PBMCs were stimulated with hIL-12 ± heparin and analyzed as described in Supplementary Methods. (a) Gating strategy for analysis of intracellular IFN-γ expression. IFN-γ positive populations were determined based on background production in cells that were not stimulated with IL-12. (b) PBMC subset analysis revealed that natural killer cells are the primary responders to hIL-12+heparin stimulation. NK: natural killer cells; NKT: natural killer T-cells; PBMCs: peripheral blood mononuclear cells; IL-12: Interleukin-12; IFNγ: Interferon-gamma.

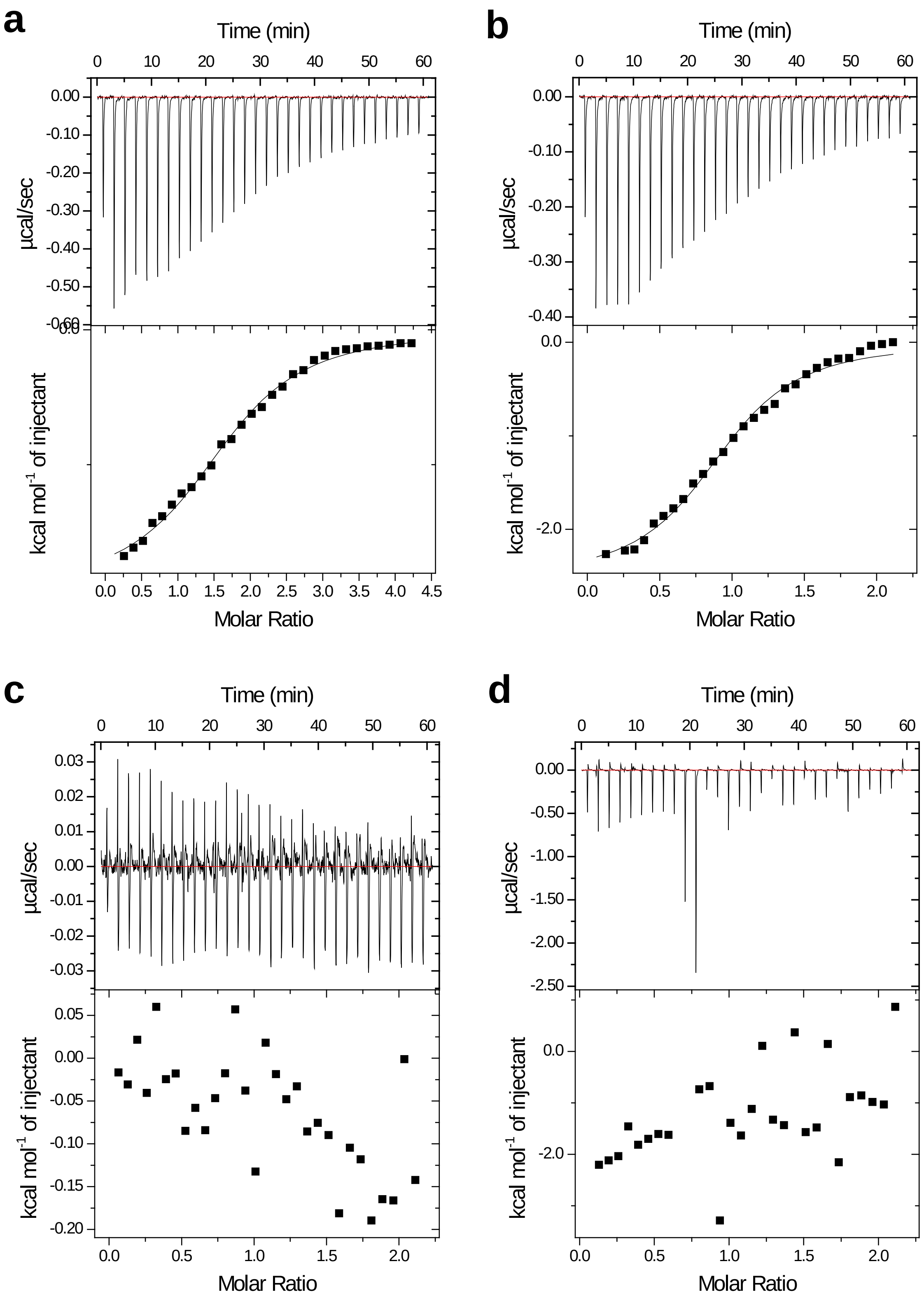
a
b
c
d
Time (min)
µcal/sec
kcal mol-1 of injectant
Molar Ratio
0 10 20 30 40 50 60
0.00 -0.10 -0.20 -0.30 -0.40 -0.50 -0.60
0.0 0.5 1.0 1.5 2.0 2.5 3.0 3.5 4.0 4.5
-2.0
0.03 0.02 0.01 0.00 -0.01 -0.02 -0.03
0.05 0.00 -0.05 -0.10 -0.15 -0.20
-0.50 -1.00 -1.50 -2.00 -2.50

e

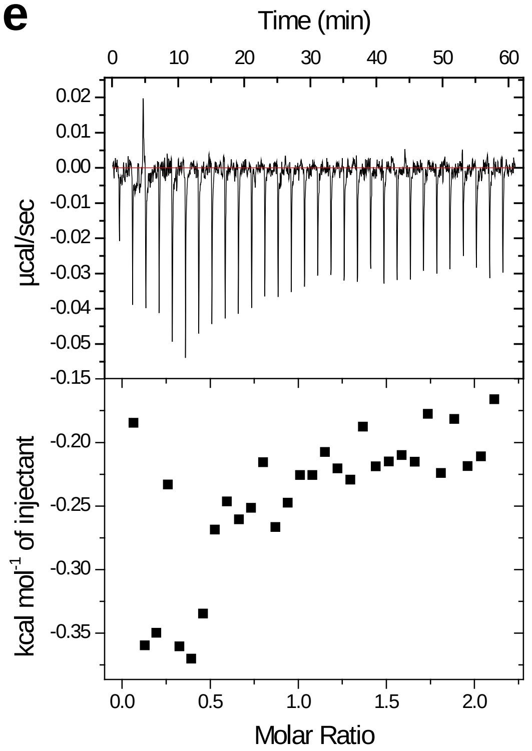

Figure S2. Isothermograms for hIL-12 interactions with various GAGs. Isothermograms describe binding interactions between hIL-12 and (A) heparin, (B) heparin sulfate, (C) chondroitin sulfate, (D) hyaluronic acid, or (E) dextran. The upper panel of each isothermogram shows the raw data obtained for each of the 30 injections. The lower panels display the best fit data to one-set of sites binding model using Origin™ v7.0 software.

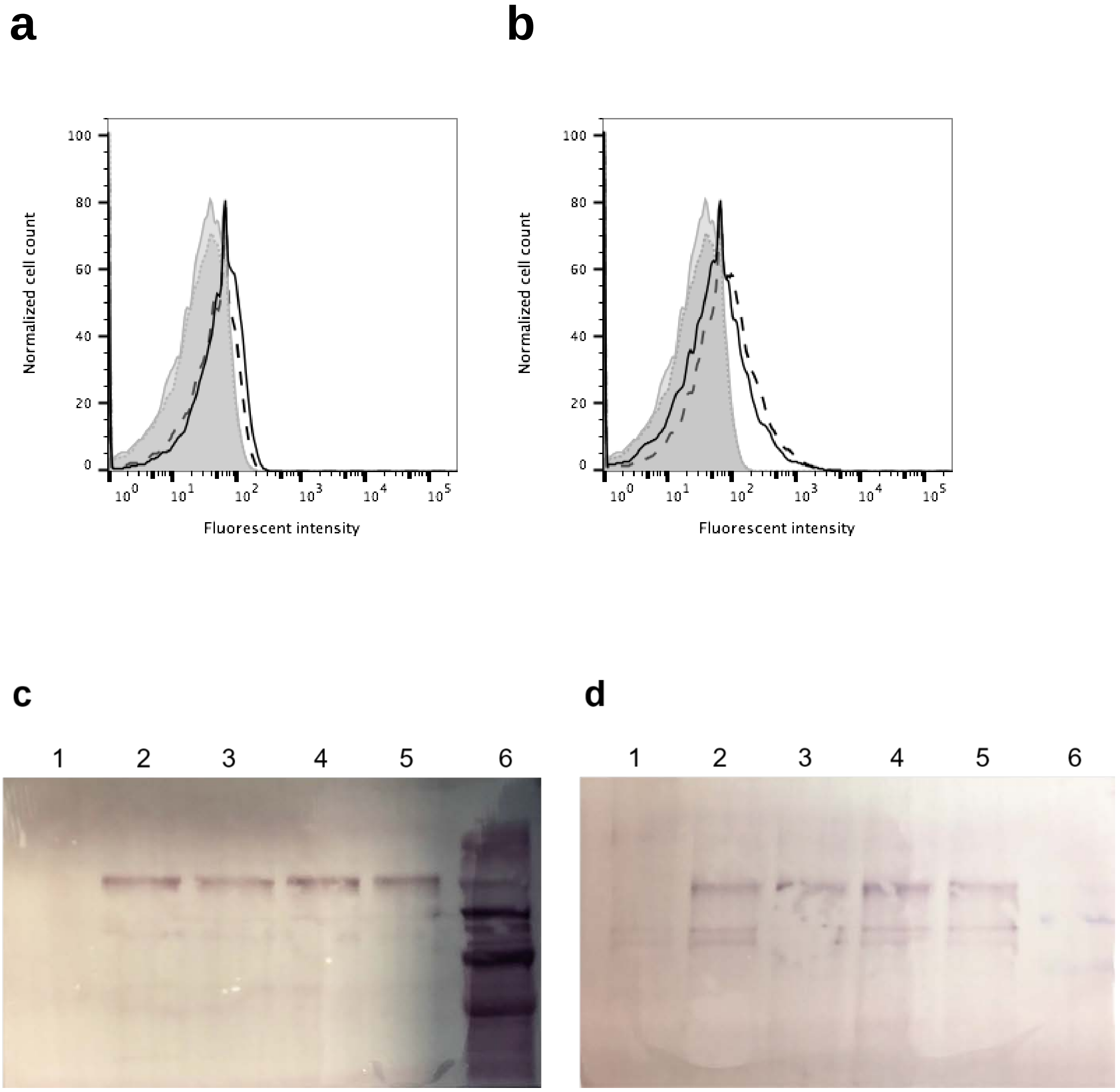

Figure S3. Analysis of IL12R expression in mutant and wild-type NK92MI cells. IL12Rβ1^{mut}/IL12Rβ2^{mut} and wild-type NK-91MI were stained with antibodies against IL12Rβ1 and IL12Rβ1 and analyzed via flow cytometry as indicated in Supplementary Methods. (A) IL12Rβ1 expression in wild-type NK-91MI cells (solid line) and IL12Rβ1^{mut}/IL12Rβ2^{mut} NK-92MI cells (dashed line). (B) IL12Rβ2 expression in wild-type NK-91MI cells (solid line) and IL12Rβ1^{mut}/IL12Rβ2^{mut} NK-92MI cells (dashed line). Isotype controls for wild-type NK-92MI cells (filled-solid line) and IL12Rβ1^{mut}/IL12Rβ2^{mut} NK-92MI cells (filled-dashed line) are displayed in each panel. Data are representative of three independent experiments. Western blots for IL12Rβ1 (C) and IL12Rβ2 (D) were developed as described in Supplementary Methods. *Lane 1*: HEK-293 cells; *Lanes 2-4*: IL12Rβ1^{mut}/IL12Rβ2^{mut} NK-92MI cells; *Lane 5*: wild-type NK-92MI; *Lane 6*: protein ladder.
